# Effect of Polyaryl Hydrocarbons on Cytotoxicity in Monocytic Cells: Potential Role of Cytochromes P450 and Oxidative Stress Pathways

**DOI:** 10.1371/journal.pone.0163827

**Published:** 2016-09-29

**Authors:** Sabina Ranjit, Narasimha M. Midde, Namita Sinha, Benjamin J. Patters, Mohammad A. Rahman, Theodore J. Cory, P. S. S. Rao, Santosh Kumar

**Affiliations:** 1 Department of Pharmaceutical Sciences, University of Tennessee Health Science Center, Memphis, TN, 38163, United States of America; 2 Department of Clinical Pharmacy, University of Tennessee Health Science Center, Memphis, TN, 38163, United States of America; 3 Department of Pharmaceutical Sciences, The University of Findley, 1000 N. Main Street, Findlay, OH, 45840, United States of America; University of Rochester Medical Center, UNITED STATES

## Abstract

**Background:**

Benzo(a)pyrene (BaP), naphthalene (NPh), phenanthrene (Phe), benzo(a)antharacene (BeA), and benzo(b)fluoranthene (BeF) are known carcinogenic polyaryl hydrocarbons (PAHs) present in cigarette smoke. This study was designed to examine the relative effect of these constituents on the cytotoxicity of monocytic cells and the possible mechanism of PAH-mediated cytotoxicity.

**Methods:**

We examined the acute (6–24 hours) and chronic (7 days) effects of these PAHs on the expression of cytochromes P450 (CYPs), oxidative stress, and cytotoxicity. The treated cells were examined for mRNA and protein levels of CYPs (1A1 and 3A4) and antioxidants enzymes (AOEs) superoxide dismutase-1 (SOD1) and catalase. Further, we assessed the levels of reactive oxygen species (ROS), caspase-3 cleavage activity, and cell viability. We performed these experiments in U937 and/or primary monocytic cells.

**Results:**

Of the five PAHs tested, after chronic treatment only BaP (100 nM) showed a significant increase in the expression of CYP1A1, AOEs (SOD1 and catalase), ROS generation, caspase-3 cleavage activity, and cytotoxicity. However, acute treatment with BaP showed only an increase in the mRNA expression of CYP1A1.

**Conclusions:**

These results suggest that of the five PAHs tested, BaP is the major contributor to the toxic effect of PAHs in monocytic cells, which is likely to occur through CYP and oxidative stress pathways.

## Introduction

According to the International Agency for Research on Cancer (IARC), there are around 5,300 chemicals identified in mainstream cigarette smoke, among which seventy are classified as carcinogens [1, 2]. The IARC monograph program has listed several categories of chemical compounds that are potential carcinogens in cigarettes, including polyaryl hydrocarbons (PAHs), N-nitrosamines, aldehydes, phenols, volatile hydrocarbons, and other organic and inorganic compounds. Since the identification of cigarette constituents in 1950s, several studies have been conducted with regard to its carcinogenicity in different human organs. Tobacco-specific nitrosamines and PAHs are the most studied cigarette carcinogens. Tobacco-specific nitrosamines, 4-(methylnitrosamino)-1-(3-pyridyl)-1-butanone (NNK) and N’-nitrosonornicotine (NNN) are reported to cause lung cancer and oral cavity cancer [3]. PAHs are associated with several cancers such as skin, lung, oral, and breast cancer [4–7]. Among the hundreds of PAHs present in the cigarette smoke, benzo(a)pyrene (BaP) is the most extensively studied PAH due to its known carcinogenic effects. Naphthalene (NPh) and phenanthrene (Phe) have relatively low carcinogenicity, but they contribute highly to the total PAH yield [8].

Cytochrome P450 (CYP) enzymes metabolize PAHs successively into epoxides and diol-epoxides [9]. These metabolites cause cellular damage either by forming DNA and protein adducts or by generating reactive oxygen species (ROS) [10, 11]. BaP is metabolized by CYP1A1 into a carcinogen, BaP-7,8-dihydrodiol-9,10-epoxide (BPDE), via a series of metabolic reactions. BPDE is reported to form DNA adducts which cause mutations in DNA, ultimately leading to carcinogenesis in lung and skin epithelial cells [12]. BaP is also reported to cause apoptosis in ovarian follicular cells via a CYP-mediated pathway [13]. Several reports in literature suggest the role of aryl hydrocarbon receptor-mediated CYP-induction and the subsequent oxidative stress in various forms of cancer and cardiac toxicity [14, 15]. Some studies reveal that BaP causes carcinogenic effects by inducing CYP1A1 expression through binding of p-53 to promoter region of CYP1A1 [16]. Rapid formation of [D10] *r*-1,*t*-2,3,*c*-4-tetrahydroxy-1,2,3,4-tetrahydrophenanthrene ([D10] PheT), a carcinogenic diol metabolite of Phe, occurs in smokers [17]. Oxidative stress, DNA damage, and cell toxicity were observed following treatment with NPh in cultured J774A.1 macrophages [18].

Previous studies from our lab have shown that nicotine, a major constituent of tobacco, causes oxidative stress in U937-derived macrophages through a CYP2A6-mediated nicotine metabolic pathway [19]. Our *in vitro* data was validated by an *in vivo* study in which, HIV-positive smokers displayed a higher rate of nicotine metabolism by CYP2A6 than HIV-negative smokers [20]. The results from these studies were consistent with the findings from another study, in which there was an increase in viral load, cytokines, and oxidative stress, likely through CYP pathway, in the plasma and monocytes of HIV-infected smokers compared to HIV-infected nonsmokers [21]. As the ultimate goal of the aforementioned studies was to explore the role of CYP enzymes in smoking-induced oxidative stress and HIV-1 pathogenesis, the experiments were conducted in monocytes/macrophages. Monocytes/ macrophages are one of the cellular targets for HIV-1 and they also serve as important viral reservoirs [22]. Smoking may enhance the infiltration of the infected monocytes/macrophages into the brain and further infect the microglia and astrocytes, ultimately leading to NeuroAIDS. However, it is still not clear whether other cigarette components besides nicotine are also responsible for CYP-mediated oxidative stress and HIV-1 replication in monocytes/macrophages. Therefore, the current study was designed to first examine the relative contribution of five PAHs; BaP, NPh, Phe, BeA, and BeF, on the regulation of CYP enzymes, oxidative stress, and cytotoxicity in U937 monocytic cells followed by primary macrophages.

## Materials and Methods

### Cell culture and treatment

#### U937 monocyte cells

The U937 monocytic cell line used for the study was obtained from ATCC (Manassas, VA). The cells were cultured in Roswell Park Memorial Institute (RPMI) 1640 media (Sigma Aldrich, St. Louis, MO), which included 1% gentamycin (Mediatech Inc. Manassas, VA), L-glutamine (Fischer Scientific, PA), and sodium bicarbonate (Fischer Scientific, PA). To assess the acute effect of PAHs (1 μM NPh, 1 μM Phe, and 100 nM BaP) at 3, 6, and 9 hours, 0.8 million cells/well were seeded in a 12-well plate. The cells were incubated overnight at 37°C in an incubator with 5% CO_2_ prior to treatment with PAHs. Following treatment, cells were collected at the designated time points. To assess the chronic effect of PAHs (100 nM NPh, 100 nM Phe, 100 nM BeA, 100 nM BeF, 5 nM BaP, 25 nM BaP, and 100 nM BaP), 0.1 million cells were seeded per well in a 6-well plate. The cells were treated with PAHs after 30 minutes of incubation. The cells were treated every 12 hours for 7 days, with addition of 250 μL of fresh media during each treatment to maintain the concentrations of PAHs. DMSO treated cells served as control for both the acute and chronic treatment paradigms.

#### Primary macrophages

Blood from interstate blood bank Inc. (Memphis, TN) was diluted with phosphate-buffered saline (PBS, Life Technologies, NY), layered on Ficoll (Mediatech Inc. Manassas, VA) and centrifuged at 400g for 30 minutes. The white ring of peripheral blood mononuclear cells (PBMCs) formed in between the plasma and Ficoll layers were carefully isolated. The PBMCs were washed with PBS several times to ensure the removal of Ficoll. The cells were incubated with ammonium-chloride-potassium (ACK) lysing buffer (Life Technologies, NY) at 4°C for 15 minutes to lyse and remove any red blood cells, if present. The clear pellets of PBMCs were then cultured in RPMI media with human serum and macrophage colony-stimulating factor for macrophage differentiation. The differentiated macrophages were treated with BaP (100 nM) for 6 days, every 24 hours with a addition of 500 μl fresh media after each treatment.

### Isolation of DNA, RNA, and protein

DNA, RNA, and protein were isolated from the lysed cells using Allprep DNA/RNA/Protein QIAGEN Kit (Valencia, CA) using the supplier’s protocol. RNA and DNA were quantified using Nanodrop 2000c UV-Vis Spectrophotometer (Thermo Fischer Scientific, Rockford, IL) by measuring their absorbance at 260 nm. The protein was quantified using the BCA protein assay kit (Thermo Fischer Scientific, Rockford, IL).

### Quantitative reverse transcriptase polymerase chain reaction (RTPCR)

Quantitative RTPCR was performed to measure the relative mRNA fold expression of the CYPs 1A1, 3A4 and the antioxidant enzymes (AOEs) superoxide dismutase 1 (SOD1) and catalase in U937 cells upon exposure to PAHs. Purified RNA (120 ng) was reverse transcribed to cDNA using a SimpliAmp Thermal Cycler (Applied Biosystems, Foster City, CA). The cDNA was amplified in a Step-One Plus Real-Time PCR System (Applied Biosystems, Foster City, CA) using TaqMan Gene Expression kit (Applied Biosystems, Foster City, CA). The following probes from Applied Bioscience were used for the Q-RTPCR reaction: CYP1A1 (Hs01054794_m1), CYP3A4 (Hs00430021_ml), SOD1 (Hs00533490_ml) and catalase (Hs00156308_ml). The 2^-ΔΔCt^ method was used to calculate the relative mRNA fold expression of the genes, using glyceraldehyde 3-phosphate dehydrogenase (GAPDH) as an endogenous control.

### Western blotting

To determine the expression of proteins of interest, 30 μg of proteins in 5% SDS were separated on a polyacrylamide gel (4% stacking, 10% resolving gel) at 150 V for 70 minutes. The proteins from the gel were transferred to a polyvinylidene fluoride membrane at 0.35 Amp for 90 minutes. The transferred blots were blocked with 5–10 mL of Li-Cor blocking buffer (LI-COR Biosciences, Lincon, NE) for 1 hour and incubated overnight with primary antibodies (GAPDH Rabbit Mab, 1:2000 dilution, Cell Signaling Technology, Danvers, MA; CYP1A1 rabbit Mab, 1:200 dilution, Abcam, Cambridge, MA; CYP3A4 Mouse Mab. 1:200 dilution, Santa Cruz Biotechnology. Inc. Dallas, TX; SOD1 Mouse Mab, 1:1500 dilution, Santa Cruz Biotechnology. Inc. Dallas, TX; Catalase Mouse Mab, 1:1200 dilution, Santa Cruz Biotechnology. Inc. Dallas, TX) at 4°C. After subsequent washing, the blots were incubated with corresponding secondary antibodies (1:10000 dilution, Goat anti-Mouse Mab, LI-COR Biosciences, Lincon, NE; 1:10000 dilution, Goat anti-Rabbit Mab, LI-COR Biosciences, Lincon, NE) for 1 hour at room temperature. The blots were scanned with Li-Cor Scanner (LI-COR Biosciences, Lincon, NE) and the densitometry data obtained from Image Studio Lite version 4.0 were used to calculate the fold expression of the proteins. GAPDH was used as an internal loading control to normalize the expression of sample proteins.

### Measurement of reactive oxygen species (ROS) and cell viability

ROS and cell viability were quantified by flow cytometry using the fluorescence dye 5-(and-6)-chloromethyl 2’,7’- dichlorodihydrofluorescein diacetate (CM-H_2_DCFDA) (Life Technologies, Oregon, USA) and Ghost Dye (Tonbo Biosciences, San Diego, CA), respectively. The treated cells were thoroughly washed with PBS and resuspended in 1 mL of PBS containing 2% FBS supplemented with 2–5 μL of CM-H_2_DCFDA and 1 μL of the Ghost dye. The cells were then incubated at room temperature in the dark for 30 minutes and subsequently washed and resuspended in 300 μL of PBS containing 2% FBS. Dichlorodihydrofluorescein (DCF) emission at 525 ± 20 nm, which is proportional to the ROS generated in the cells, and emission of Ghost dye at 780 nm, which is proportional to the cell viability, were detected by flow cytometry (BD Biosciences, San Jose, CA). The data were analyzed by using the BD FACS software version 8.

### XTT assay

Cell viability was also measured using the XTT assay kit (Cell Signaling Technology Inc., Danvers, MA). The PAH-treated cells (0.12 million) were suspended in 200 μL of phenol red-free media. XTT detection solution was made by mixing electron coupling solution and XTT reagent in the ratio of 1:50. Fifty microliters of the XTT detection solution was added to each well of the 96-well plate containing 200 μL of the cell suspension and the plate was incubated at 37°C for three hours. The absorbance measured at 450 nm represented cell viability.

### Caspase-3 activity assay

Caspase-3 activity was measured using Caspase-3 colorimetric assay kit (BioVision, Inc., Milpitas, CA). Cells (1–5 millions) obtained after the treatments were lysed and protein was extracted. About 100 μg of the protein was diluted to a final volume of 50 μl in cell lysis buffer. Each protein sample was added to 50 μl of 2X reaction buffer (containing 10 mM DTT) and 5 μl of 4 mM DEVD-pNA substrate and the reaction mixture was incubated at 37°C for 1 hour. Absorbance of the samples was measured at 405 nm.

### Statistical analysis

For the statistical analysis of the data obtained from RTPCR, Western blot, cell viability assay, ROS level, and caspase-3 activity assay, mean ± SEM was calculated and One-way ANOVA was applied to calculate p values. A p value of ≤0.05 was considered significant.

## Results

### Effect of chronic treatment of BaP, NPh, and Phe on the expression of CYPs at mRNA and protein levels

**Fig 1:**
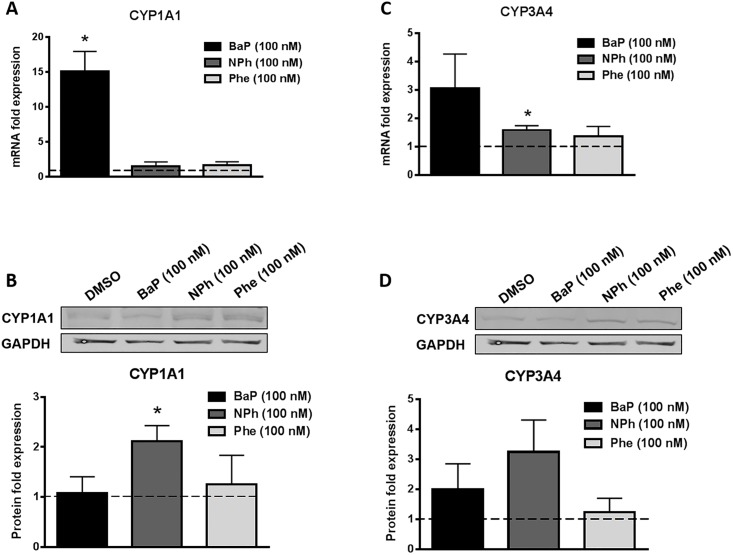
Effect of chronic (7 days) treatment of BaP, NPh, and Phe on mRNA and protein expression of CYP1A1 (A-B) and CYP3A4 (C-D) in U937 cells. The U937 cells were treated with 100 nM BaP, 100 nM NPh, and 100 nM Phe for seven days. The mRNA fold expressions were calculated using qRT-PCR and normalized with control (DMSO treated cells) whose expression was set at 1-fold. GAPDH was used as an endogenous control. The protein fold expressions were quantified by Western blot and normalized with control that was set to 1-fold at every time point. Blots are representative of at least three independent experiments. The data are presented as a mean ± SEM of three independent experiments. *represents p ≤ 0.05, compared with the control group.

PAHs are metabolized by various CYP enzymes, mainly CYPs 1A1, 1B1, and to a lesser extent by CYPs 1A2, 2C9, 3A4, and 2C19 into reactive metabolites and produce ROS that cause DNA damage [23]. Therefore, to examine the effect of these compounds on expressions of CYPs 1A1 and 3A4 that are prominently present in U937 cells, we treated the cells separately with 100 nM of each compound for seven days and measured the mRNA and protein levels of CYP1A1 and CYP3A4 (Fig 1). With BaP treatment, we observed ~15 fold increase (p≤0.05) in the CYP1A1 mRNA expression (Fig 1A) and ~3 fold increase in CYP3A4 mRNA expression (Fig 1C). However, the CYP1A1 and 3A4 protein expression levels quantified from the Western blots did not correspond to the respective changes in mRNA expression levels. NPh and Phe did not show much change in the induction of either CYPs. Interestingly, there was ~2.5 fold (p≤0.05) increase in the protein expression of CYP1A1 in the cells treated with NPh (Fig 1B).

### Effect of chronic treatment of BaP, NPh, and Phe on the expression of AOEs at the mRNA and protein levels

**Fig 2:**
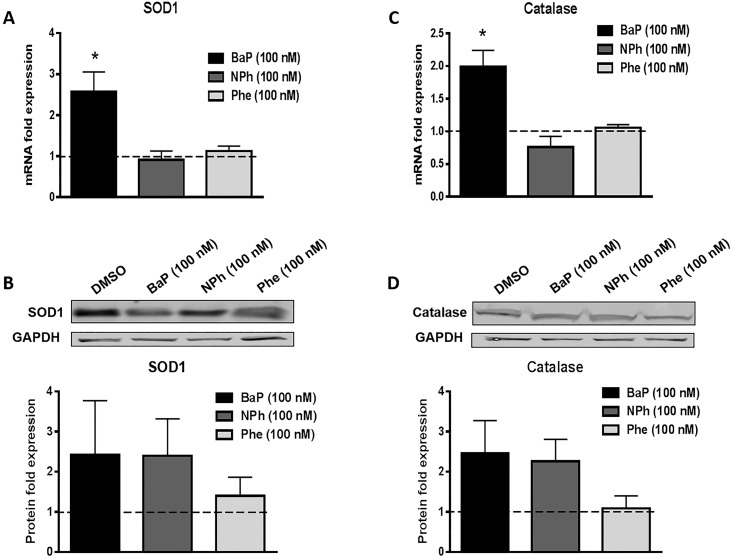
Effect of chronic (7 days) treatment of BaP, NPh, and Phe on mRNA and protein expression of AOEs SOD1 (A-B) and catalase (C-D) in U937 cells. The U937 cells were treated with 100 nM BaP, 100 nM NPh, and 100 nM Phe for seven days. The mRNA fold expressions were calculated using qRT-PCR and normalized with control (DMSO treated cells) whose expression was set at 1-fold. GAPDH was used as an endogenous control. The protein fold expressions were quantified by Western blot and normalized with control (DMSO treated cells) whose expression was set at 1 fold. Blots are representative of at least three independent experiments. The data are presented as a mean ± SEM of three independent experiments. *represents p ≤ 0.05, compared with the control group.

PAHs are likely to induce expression of AOEs to combat PAH-induced oxidative stress in U937 cells. To determine whether these compounds alter the expression of AOEs, we measured the mRNA and protein levels of the major AOEs SOD1 and catalase (Fig 2). Chronic (seven days) exposure of 100 nM BaP upregulated the mRNA expression of both SOD1 (~2.5 fold, p≤0.05, Fig 2A) and catalase (~2 fold, p≤0.05, Fig 2C). The corresponding protein expression levels of both AOEs were also upregulated by similar proportions: SOD1 (~2.5 fold) and catalase (~2.5 fold) but the data were not statistically significant. Further, treatment with NPh or Phe did not significantly alter the mRNA or protein expression of AOEs.

### Effect of chronic treatment of BaP, NPh, and Phe on ROS and cell viability

**Fig 3:**
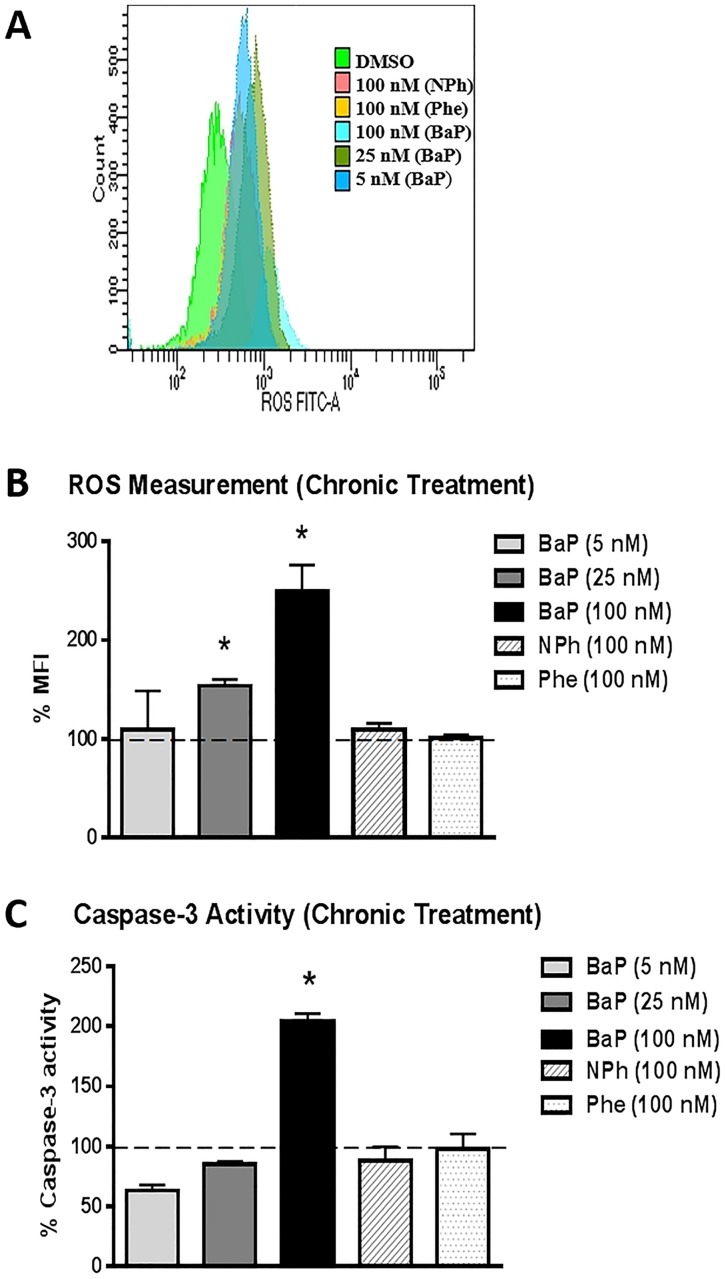
Effect of chronic (7 days) treatment of BaP, NPh, and Phe on Reactive Oxygen Species (ROS) (A-B) and caspase-3 activity (C) in U937 cells. The U937 cells were treated with 5 nM BaP, 25 nM BaP, 100 nM BaP, 100 nM NPh, and 100 nM Phe for seven days. ROS level and caspase-3 activity were calculated and normalized with control that was set as 100%. The data is presented as mean ± SEM of the three independent experiments. * represents p ≤ 0.05, compared with the control group.

**Fig 4:**
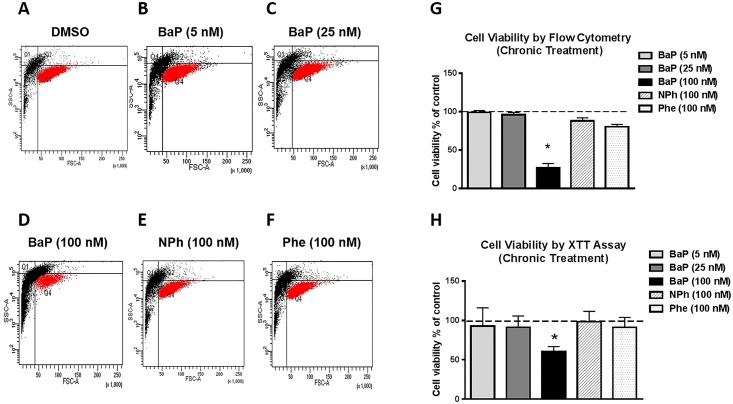
Cell viability upon chronic treatment by BaP, NPh, and Phe measured by flow cytometry (A-G) and XTT assay (H). The U937 cells were treated with 5 nM BaP, 25 nM BaP, 100 nM BaP, 100 nM NPh, and 100 nM Phe for seven days. Cell viability was calculated using flow cytometry and XTT assay, and normalized with control that was set as 100%. The data is presented as mean ± SEM of the three independent experiments. * Represents p ≤ 0.05, compared with the control group.

The cells were treated with three different concentrations of BaP (5 nM, 25 nM, and 100 nM), 100 nM NPh, and 100 nM Phe for seven days to examine the chronic effect of the PAHs on ROS generation and cell viability. A significant concentration-dependent increase in ROS was observed with the chronic treatment of 25 nM BaP (~1.5 fold, p≤0.05) and 100 nM BaP (~ 2.5 fold, p≤0.05) (Fig 3A and 3B). However, there was no significant alteration in the ROS levels with 5 nM BaP. Similarly, no significant change in ROS was observed with NPh and Phe treatments. A statistically significant (p≤0.05) decrease in cell viability (~60%) was observed with 100 nM BaP treatment. Lower concentrations (5 nM and 25 nM) of BaP, as well as, the 100 nM NPh and Phe treatments, however, did not have any effect on cell toxicity (Fig 4G). Similar decrease in cell viability with 100 nM BaP treatment was observed using XTT cell viability assay (~40% decrease in cell viability, Fig 4H). Fig 4A–4F show the graphical representation of cell viability, measured via flow cytometry.

### Effect of chronic treatment of BaP, NPh, and Phe on caspase-3 activity

In order to delineate the mechanism of cytotoxicity after chronic treatment of PAHs (BaP, NPh, and Phe), we monitored the caspase-3 activity. We observed a significant increase in caspase-3 activity with 100 nM BaP treatment (~2 fold, p≤0.05, Fig 3C). However, there was no effect on caspase-3 activity with lesser concentrations of BaP (5 nM and 25 nM) or 100 nM of NPh or Phe.

### Effect of chronic treatment of BeA and BeF on ROS, cell viability, and caspase-3 activity

**Fig 5:**
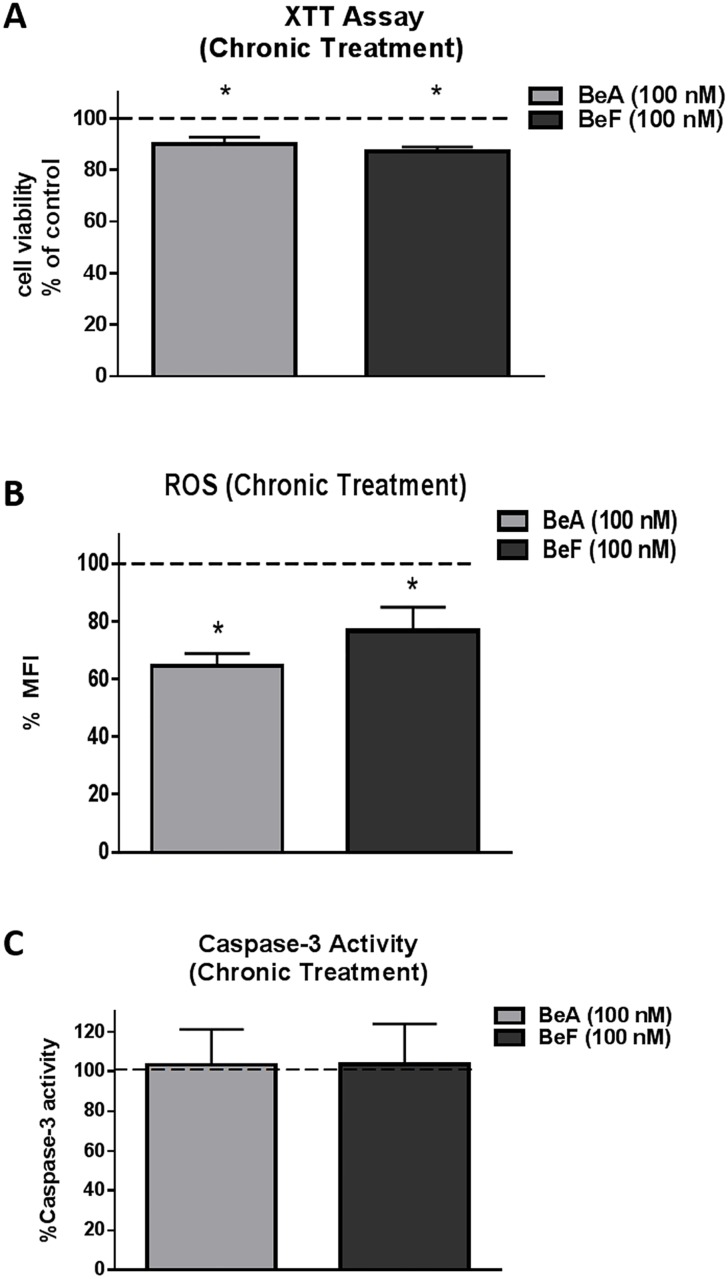
Cell viability (A), Reactive Oxygen Species (ROS) level (B) and caspase-3 activity (C) upon chronic treatment by BeA and BeF in U937 cells. The U937 cells were treated with 100 nM BeA and 100 nM BeF for seven days. ROS level, cell viability, and caspase-3 activity were measured and normalized with control that was set as 100%. The data is presented as mean ± SEM of the three independent experiments. * represents p ≤ 0.05, compared with the control group.

In addition to three PAHs (BaP, NPh, and Phe), BeA and BeF are other PAHs that have carcinogenic potential. Therefore, we also studied these compounds with regard to oxidative stress and cytotoxicity. The cells were treated with 100 nM of BeA or BeF for seven days and were examined for ROS level and cell viability. Cell viability data from XTT assay showed approximately 10–13% cell death when treated with 100 nM of BeA or BeF for seven days (Fig 5A). A significant decrease in ROS level was observed with the chronic treatment of BeA 100 nM (~35%, p≤0.05) as well as BeF 100 nM (~23%, p≤0.05) (Fig 5B). There was no significant change in the caspase-3 activity with chronic treatment of both the compounds (Fig 5C). Overall, treatment with BeA and BeF had relatively less effect on the oxidative stress and cell viability of U937 compared to BaP.

### Effect of chronic treatment of BaP on CYP expression, ROS and cell viability in primary macrophages

**Fig 6:**
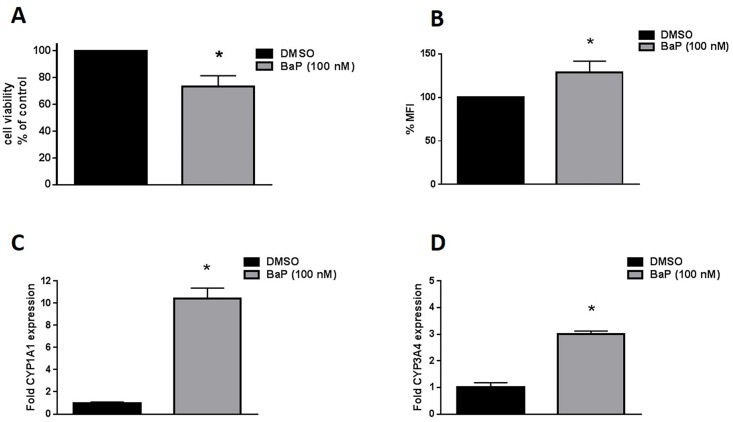
Cell viability (A), Reactive Oxygen Species (ROS) level (B) and mRNA expression level of CYP1A1 (C) and CYP3A4 (D) upon chronic treatment by BaP in primary macrophages. The differentiated macrophages obtained from healthy donors were treated with BaP (100 nM) for 6 days. Cell viability and ROS were calculated were calculated and normalized with control that was set as 100%. The mRNA expression of treated cells were normalized with control (DMSO treated cells) whose expression was set at 1-fold. The results for cell viability were confirmed using 3 different donors. Due to inadequate number of primary cells, ROS and CYPs expression level were examined in only one donor. The data is presented as mean ± SEM of the three replicates for ROS and two replicates for CYP expression levels. * represents p ≤ 0.05, compared with the control group.

Of the five compounds, only exposure with BaP (100 nM) resulted in significant increased expression of CYPs and AOEs, ROS and cell death in U937 cells. So, we exposed the primary macrophages with 100 nM BaP to verify our observations in U937 cells. When we treated the primary macrophages with BaP 100 nM for seven days, we observed ~25% cell death accompanied by ~30% increase in ROS and ~10 and ~ 3 fold (p≤0.05) increase in CYP 1A1 and 3A4 respectively (Fig 6A–6D). These results confirm that BaP exposure leads to cytotoxicity in monocytes/macrophages via CYP-mediated oxidative stress pathway.

### Effect of acute treatment of BaP, NPh, and Phe on CYP and AOE expression, ROS and cell viability in U937 cells

**Fig 7:**
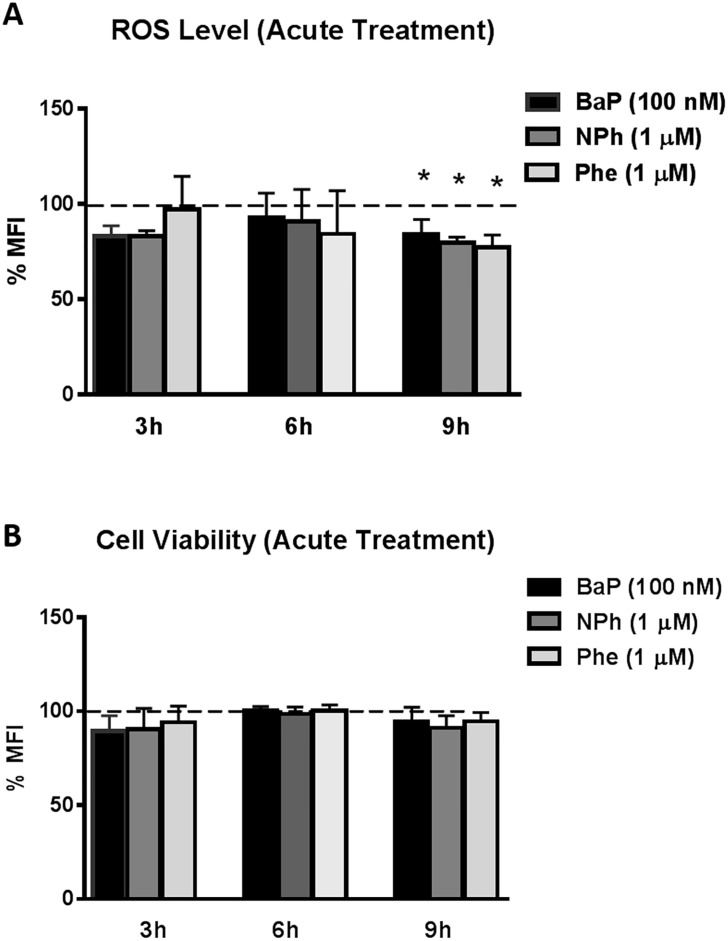
Effect of acute treatment of BaP, NPh, and Phe on Reactive Oxygen Species (ROS) (A) and Cell viability (B) in U937 cells. The U937 cells were treated with 100 nM BaP, 1 μM NPh, and 1 μM Phe for 3, 6, and 9 hours. ROS level and cell viability were measured using flow cytometry. Measured values at every time point were normalized to control that was set at 100%. X-axis and Y-axis correspond to time points and % of mean fluorescent intensity (%MFI), respectively. The data are presented as a mean ± SEM of three independent experiments. * represents p ≤ 0.05 compared with the control group.

In addition to chronic treatment, we also monitored the effect of the acute exposure of BaP, NPh, and Phe in U937 cells. The cells were treated with BaP (100 nM), NPh (1 μM), and Phe (1 μM) for 3, 6, and 9 hours. There was no significant change in ROS and cell viability in cells treated with the PAHs for 3 and 6 hours (Fig 7A and 7B). However, a significant decrease (p≤0.05) of ~10% in ROS was observed with all the three compounds after 9 hours of exposure. The cell death at 9 hours was not particularly different from the control. The small decrease in ROS would not have been sufficient enough to cause a significant impact on cell viability. In general, there was minimal to no effect of acute exposure of BaP, NPh, and Phe on oxidative stress and cytotoxicity in U937 cells.

Furthermore, there was no significant effect of the acute exposure of the PAHs over the induction of CYPs and AOEs (S1–S4 Figs). Only BaP exposure showed a significant ascending trend in the mRNA expression of CYP1A1 over time (~10 fold increase at 6 hours to ~30 fold increase at 24 hours, p≤0.05), as well as with increasing concentration (S1A Fig). However, there was no significant increase in the expression of CYP1A1 protein with either 100 nM or 1 μM BaP treatment (S1B Fig). None of the PAHs showed significant change in either the mRNA and protein expression levels of CYP3A4, except for the treatment with Phe (1 μM), which showed ~3 fold increase in CYP3A4 expression at mRNA level at 12 hours (p≤0.05) (S2 Fig). At 24 hours, we observed a significant increase in the SOD1 mRNA expression level: ~3 fold with (1 μM) BaP (p≤0.05) and ~2 fold with 100 nM NPh (p≤0.05). None of these compounds showed significant alteration in the protein expression levels of SOD1 (S3B, S3D and S3F Fig). Similarly, no significant change in the mRNA and protein expression levels of catalase was observed with any of the acute PAH treatments (S4 Fig).

## Discussion

Carcinogenic effects of smoking have been widely studied in various organs, especially lungs and liver [24, 25]. Several studies have also reported CYP-mediated PAH toxicity in different organs [26, 27]. However, relatively less information is available on the effects of smoking constituents on the bloods cells such as monocytes and lymphocytes. A recent study conducted in our lab has shown that nicotine causes oxidative stress in monocytic cells U937 cells through a CYP-mediated pathway [19]. However, there is no report on the effect of PAHs and relative contribution of different PAHs on oxidative stress and cytotoxicity, and underlying mechanism in the monocytic cells. The present study is the first report of relative contribution of five PAHs (BaP, NPh, Phe, BeA, and BeF) on the expression of CYPs and AOEs, induction of ROS, and cytotoxicity in U937 monocytic cells. The results from this study support the existing literatures that among the tested PAHs, BaP is the most harmful compound, which causes oxidative stress and subsequent cytotoxicity, at least in part through CYP pathway.

Zhu et al. (2014) have shown that BaP (5 μM) significantly increases the expressions of CYP1A1 and CYP1B1, ROS level, and cytotoxicity in lung epithelial cells (BEAS-2B cells) after 24 hour treatment [28]. In this paper, we examined the effect of both acute (6–24 hours) and chronic (7 days) exposure of PAHs in U937 cells. We initially treated the cells with 100 nM and 1 μM of each of the PAHs for acute study. These concentrations are very near to the physiological concentrations of PAHs that caused toxicity in different cell lines [28, 29]. For the chronic treatment, the lower concentration (100 nM) was preferred because higher concentration (1 μM) resulted in immense cell death when exposed for a prolonged period. There are very few reports that account for the toxicity of chronic BaP exposure in human cell lines. For examples, reports of chronic BaP exposure in animal model suggest its association with neurotoxicity [30], DNA damage [31], carcinogenicity, and cytotoxicity [32]. Most of the studies of BaP toxicity have been conducted at higher concentration for an acute period. In this context, our experimental design to study the acute as well as chronic effects of near physiological concentrations of PAHs on monocytic cells is pragmatic because it closely simulates the effects of tobacco on these cells *in vivo*.

We have used U937 cells derived from histiocytic lymphoma tissues, which have functionally deficient p53 tumor suppressor gene due to gene mutation [33]. The phenomenon of apoptosis is apparently ceased in these cell lines, causing massive cell proliferation. However, apoptosis may occur in U937 cells when triggered by various stress factors such as ROS, via a p53-independent mechanism. There are also reports that associate p53 with BaP-induced CYP1A1 expression, which occur via p53 binding to a p53RE in the CYP1A1 regulatory region [34]. Some studies suggest that p53 upregulates the gene associated with antioxidant activity and thereby prevent the genome from oxidative damage by ROS [35]. Due to the lack of functional p53 in U937 cells, induction of CYP1A1 and antioxidants by BaP occur via a p53 independent pathway, probably via Nf-KB-mediated pathway. Another possible pathway for induction of CYPs could be via binding of BaP to arylhydrocarbon receptor (AhR) and the subsequent gene activation by constitution of the Arnt protein-BaP complex with XRE responsive element [36].

With acute treatment of three PAHs BaP, NPh, and Phe, we did not observe any significant change in the expressions of CYPs and AOEs, ROS level, or cell viability. So, we exposed the cells for seven days with 100 nM of each of the compounds to monitor their chronic effect. Out of five PAHs (BaP, NPh, Phe, BeA, and BeF), only BaP (100 nM) significantly increased the expressions of CYPs and AOEs, generation of ROS, and cytotoxicity in U937 cells. The prolonged exposure of BaP to the cells probably caused the accumulation of ROS, via CYP-pathway. Elevated ROS then contributed to the oxidative stress and cytotoxicity. An increased level of AOE expression with chronic BaP treatment is also consistent with the finding that there was an increase in the ROS level.

BaP is considered a prototype compound for PAH carcinogenicity. IARC has listed BaP as a group I human carcinogen, while NPh, Phe, BeA, and BeF have been classified as compounds possibly carcinogenic to humans (IARC 2004). BaP-induced carcinogenesis has been studied extensively for more than five decades. Previous studies reveal that CYP metabolites of BaP form DNA adducts that inactivate the tumor suppressor p53 in human epithelial cells and bronchial epithelial cells [24]. Kucab et al. (2015) has also suggested the role of BaP and its metabolites in p53 mutagenesis, which is a common pathway observed in almost half of human cancers [37]. However, the role of BaP and other PAHs with respect to oxidative stress and cytotoxicity in monocytic cells is not known. In this context, evaluation of the role of PAHs and their relative contributions to the expressions of CYPs, induction of oxidative stress, and cytotoxicity further validates the literature that BaP is toxic to many cells including monocytic cells.

BaP induces the expression of several CYPs including 1A1 and 1B1 in different cells [38–40]. Since the basal expression of CYP1B1 in U937 cells is very low [41], we examined the effect of PAHs on the expression of CYP1A1 only. Cytotoxicity induced by CYP1A1 metabolites of BaP has been reported in different cellular systems: lung cells [42], porcine urinary bladder epithelial cells [43], and bone marrow cells [44]. CYP1A1 metabolizes BaP into BPDE which forms DNA adducts leading to genotoxicity and carcinogenesis. Some studies also suggest that ROS generated from BaP via CYP pathway causes oxidative stress that lead to cytotoxicity. In the present study, CYP1A1 mRNA expression was consistently upregulated (~15 fold) following the acute and chronic treatment of BaP (100 nM) in U937 cells. However, the CYP1A1 protein levels were not consistent with the mRNA expression levels. Similar contradiction was reported in earlier studies [45], the reason for which is not clear. However, it is possible that CYP1A1 is relatively less stable upon extraction or there may be post-translational modification of the protein. The metabolites obtained from the CYP1A1-mediated oxidation of BaP may be responsible for the elevated ROS level and subsequent oxidative stress and cytotoxicity. Further investigation is required to confirm our hypothesis that CYP1A1 metabolizes BaP into ROS-generating metabolites that cause cytotoxicity in U937 cells. This can be done either by knocking down CYP1A1 gene or by using a selective inhibitor of CYP1A1 and treating with BaP to determine if ROS generation and cytotoxicity still occur. The chronic exposure to NPh (100 nM) showed ~ 2 fold increase in CY1A1 protein expression but there was no significant effect on AOE expression, ROS, cell viability and caspase-3 activity. Although, we observed increase in CYP1A1 protein expression to some extent, the induced protein may not have been sufficient enough to metabolize the compound.

CYP3A4 is a major drug metabolizing enzyme that metabolizes about 50% of the xenobiotics and is present in relatively high abundance compared to other CYPs, not only in hepatic cells, but also in U937 monocytic cells [45]. BaP-induced CYP3A4 upregulation at mRNA level has been observed in HepG2 liver cells and HEK-293 kidney cells [46]. Kumagai et. al also suggested that BaP enhances CYP3A4 gene expression via PXR activation in liver cells [47]. Furthermore, they suggested the possibility that the metabolites obtained through CYP1A1 metabolism could act as CYP3A4 inducers. It is important to study the expression level of CYP3A4 in context of BaP metabolism and toxicity. CYP3A4 does not metabolize the PAH parent compounds but it does convert dihydrodiols into diol epoxides. However, the rate of conversion is slower than that observed through CYP1A1 metabolism [23]. In the present study, we did not observed any significant change in CYP3A4 expression at mRNA and protein levels after acute or chronic treatment with BaP. Since the overall CYP3A4 expression was low, CYP1A1 metabolites also did not seem to contribute to CYP3A4 induction.

Cells are equipped with an antioxidant defense mechanism to counteract the oxidative stress resulting from elevated ROS [48]. Oxidative stress is alleviated either by endogenous antioxidants such as reduced glutathione (GSH) or by adaptive defense mechanism through induction of genes encoding AOEs [49]. The induction of genes encoding AOEs are regulated by nuclear factor erythroid 2- related factor (Nrf-2) signaling pathway [50]. Any perturbations in the AOE defense system that decrease the AOE expression may aid elevation in ROS level. Higher incidence of oxidative stress-induced carcinogenesis is reported in Nrf-2 knockout mice that are treated with BaP. Furthermore, if the level of ROS exceeds the threshold, the AOEs may not be able to overcome the resulting oxidative insult [21, 51]. SODs and catalase are major AOEs in majority of the cell lines. In case of acute treatment of BaP, we observed a slight decrease in ROS which may be attributed to the protective activity of the endogenous AOEs. We anticipated higher levels of ROS and AOEs with chronic treatment of the PAHs, but we observed the expected result only with BaP-treated cells. The elevation in gene transcription of SOD1 and catalase over the course of seven day treatment with BaP may not be sufficient enough to alleviate the high ROS level. Rather, an increase in the expression level of these AOEs are the indication that there is oxidative stress in the system.

In addition to BaP-mediated mutagenesis and carcinogenesis through DNA adduct formation, there are also reports of apoptosis induced by BaP metabolites in different human cell lines: Daudi B cells [52], H460 lung cancer cells [53], hepatoma HepG2 cells [54] and endometrial cancer RL95-2 cells [55], the latter two being directly associated with CYP-mediated metabolism of BaP. ROS triggers apoptosis by enhancing the permeability of the mitochondrial outer membrane and thereby leaking out pro-apoptotic proteins [56]. Pro-apoptotic proteins aid in the activation of caspases, cysteine proteases that cause cellular degradation. We observed apoptosis in U937 cells via caspase-3 dependent pathway, with chronic treatment of BaP. Our data suggests the involvement of CYP in the metabolism of BaP and generation of ROS with chronic treatment of BaP. Therefore, we conclude that BaP causes apoptosis in U937 cells through a caspase-3-dependent pathway. BaP-induced cytotoxicity, in turn, is mediated by ROS that is likely to be generated through CYP-mediated metabolism of BaP.

Our previous *in vitro* and *ex vivo* studies have suggested the role of nicotine metabolism via CYP2A6 in generating oxidative stress and HIV-1 replication in monocytic cells. The current study has established the fact that, in addition to nicotine, BaP is the major PAHs that is responsible for inducing oxidative stress, apoptosis, and cytotoxicity, perhaps through the CYP pathway in monocytic cells. The confirmation of increase in CYP expression, ROS generation and cytotoxicity mediated by chronic treatment of BaP in primary macrophages has further strengthened our findings from U937 monocytic cells. We speculate that BaP-induced oxidative stress via CYP metabolism would enhance HIV-1 replication. In fact, our study with cigarette smoke condensate (CSC), which contains nicotine and PAHs, has demonstrated an increase in HIV-1 replication in primary human monocyte-derived macrophages [21]. This is also based on the fact that BaP induced greater levels of ROS (>150%) compared to nicotine (15–20%) [19]. Taken together these findings indicate that BaP is likely the most active compound in cigarette smoke, and may generate oxidative stress leading to cytotoxicity and increased HIV-1 replication in monocytic cells. However, this has yet to be fully demonstrated using HIV-infected U937 and human primary monocytic cells.

## Supporting Information

S1 Fig Effect of acute treatment of BaP (A-B), NPh (C-D), and Phe (E-F) on mRNA and protein expression of CYP1A1 in U937 cells. The U937 cells were treated with 100 nM BaP, 100 nM NPh, and 100 nM Phe for 6, 12 and 24 hours. The mRNA fold expressions were calculated using qRT-PCR and the protein fold expressions were measured by Western blots, and normalized with control (DMSO treated cells) whose expression was set at 1-fold. GAPDH was used as an endogenous control. Blots are representative of at least three independent experiments. The data are presented as a mean ± SEM of three independent experiments. * represents p ≤ 0.05, compared with the control group.
(TIF)

S2 Fig Effect of acute treatment of BaP (A-B), NPh (C-D), and Phe (E-F) on mRNA and protein expression of CYP3A4 in U937 cells. The U937 cells were treated with 100 nM BaP, 100 nM NPh, and 100 nM Phe for 6, 12 and 24 hours. The mRNA fold expressions were calculated using qRT-PCR and the protein fold expressions were quantified by Western blots, and normalized with control (DMSO treated cells) whose expression was set at 1-fold. GAPDH was used as an endogenous control. Blots are representative of at least three independent experiments. The data are presented as a mean ± SEM of three independent experiments. * represents p ≤ 0.05, compared with the control group.
(TIF)

S3 Fig Effect of acute treatment of BaP (A-B), NPh (C-D), and Phe (E-F) on mRNA and protein expression of SOD1 in U937 cells. The U937 cells were treated with 100 nM BaP, 100 nM NPh, and 100 nM Phe for 6, 12 and 24 hours. The mRNA fold expressions were calculated using qRT-PCR and the protein fold expressions were quantified by Western blots, and normalized with control (DMSO treated cells) whose expression was set at 1-fold. GAPDH was used as an endogenous control. Blots are representative of at least three independent experiments. The data are presented as a mean ± SEM of three independent experiments. * represents p ≤ 0.05, compared with the control group.
(TIF)

S4 Fig Effect of acute treatment of BaP (A-B), NPh (C-D), and Phe (E-F) on mRNA and protein expression of catalase in U937 cells. The U937 cells were treated with 100 nM BaP, 100 nM NPh, and 100 nM Phe for 6, 12 and 24 hours. The mRNA fold expressions were calculated using qRT-PCR and the protein fold expressions were quantified by Western blots, and normalized with control (DMSO treated cells) whose expression was set at 1-fold. GAPDH was used as an endogenous control. Blots are representative of at least three independent experiments. The data are presented as a mean ± SEM of three independent experiments. * represents p ≤ 0.05, compared with the control.
(TIF)
