# Supplementary figures and images for: Effect of Polyaryl Hydrocarbons on Cytotoxicity in Monocytic Cells: Potential Role of Cytochromes P450 and Oxidative Stress Pathways
Source: PLoS One. 2016 Sep 29;11(9):e0163827. doi: 10.1371/journal.pone.0163827 (PMC5042547; doi:10.1371/journal.pone.0163827)

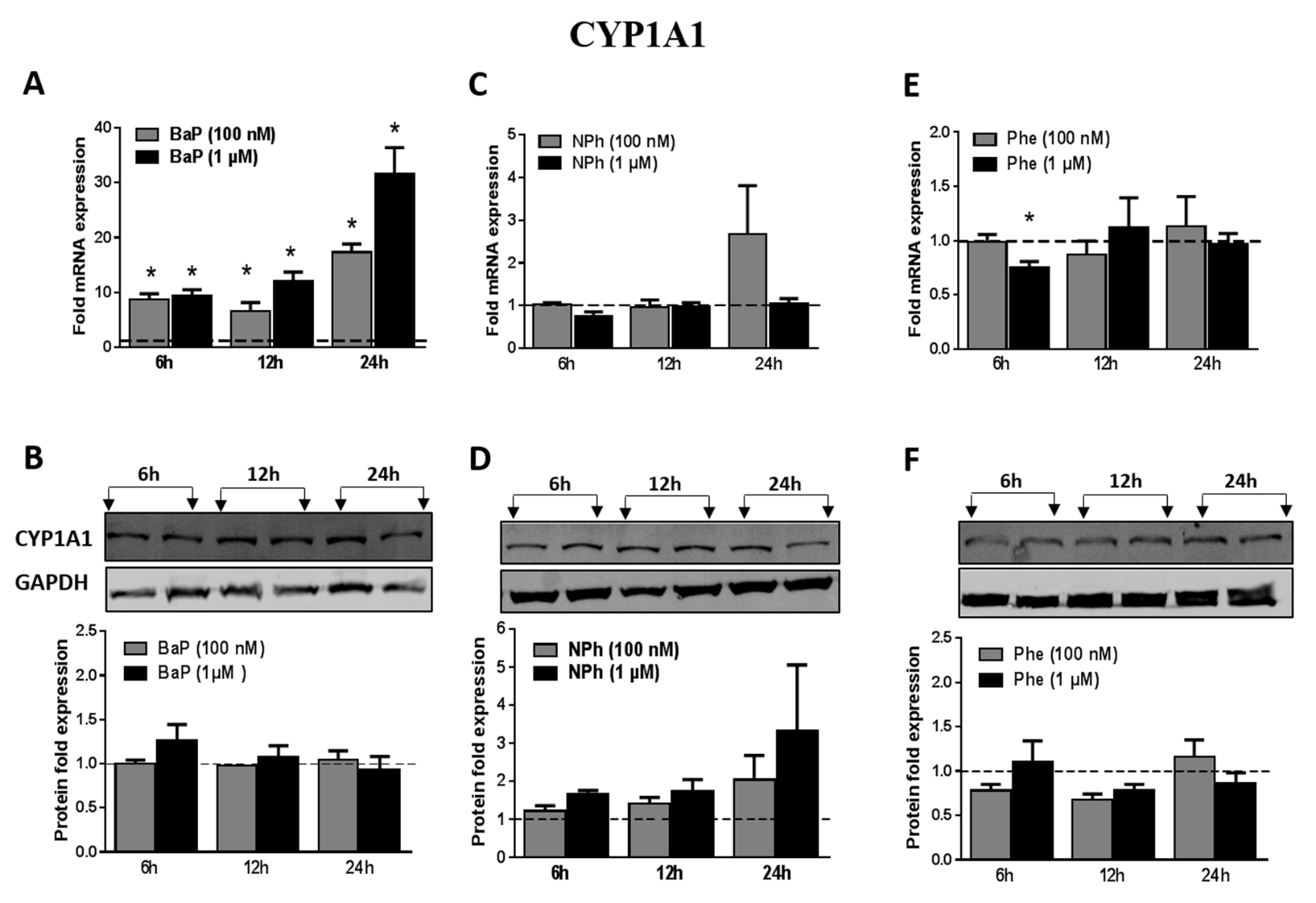

Supplement: S1 Fig — The U937 cells were treated with 100 nM BaP, 100 nM NPh, and 100 nM Phe for 6, 12 and 24 hours. The mRNA fold expressions were calculated using qRT-PCR and the protein fold expressions were measured by Western blots, and normalized with control (DMSO treated cells) whose expression was set at 1-fold. GAPDH was used as an endogenous control. Blots are representative of at least three independent experiments. The data are presented as a mean ± SEM of three independent experiments. * represents p ≤ 0.05, compared with the control group. (TIF) [file pone.0163827.s001.tif]

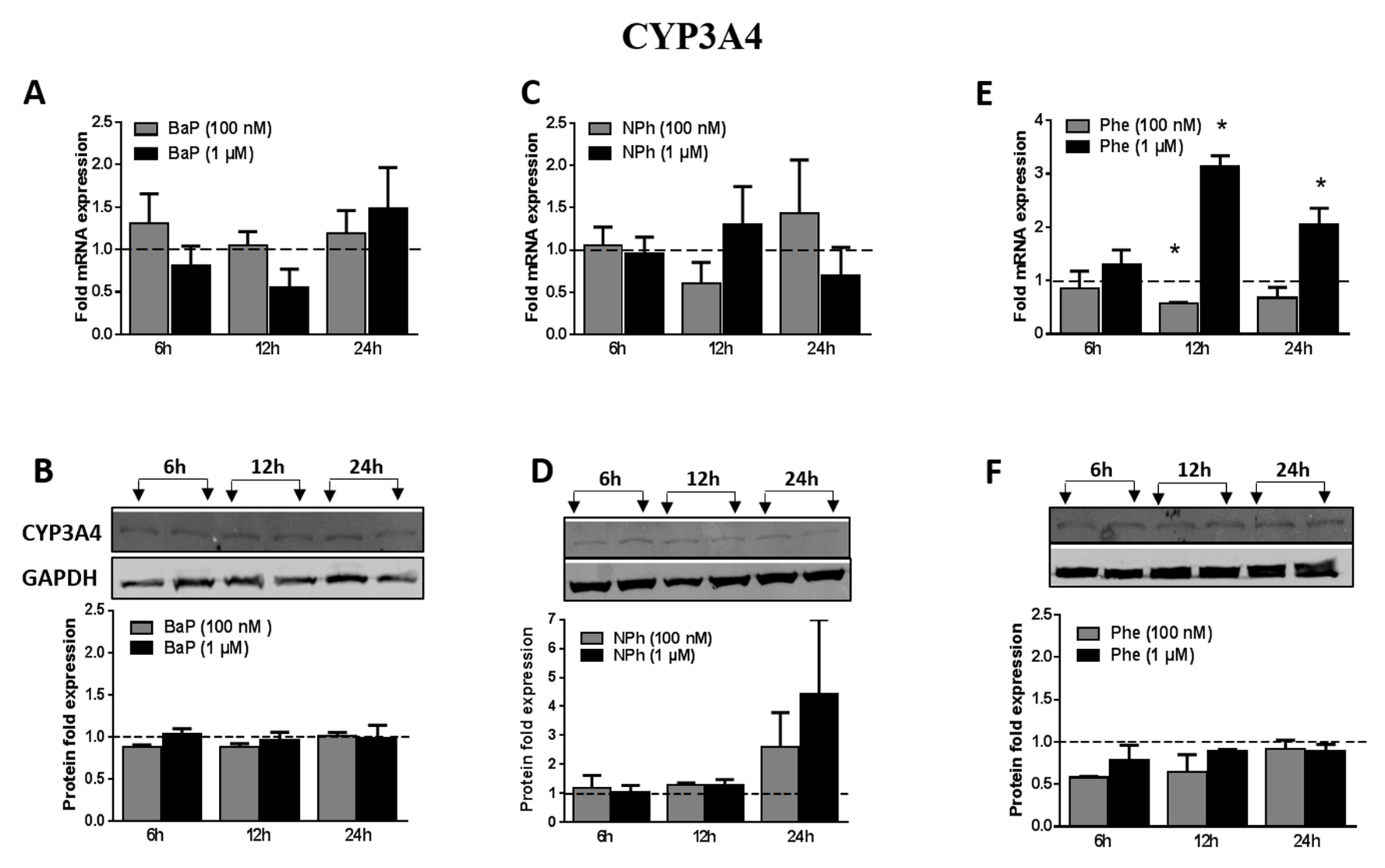

Supplement: S2 Fig — The U937 cells were treated with 100 nM BaP, 100 nM NPh, and 100 nM Phe for 6, 12 and 24 hours. The mRNA fold expressions were calculated using qRT-PCR and the protein fold expressions were quantified by Western blots, and normalized with control (DMSO treated cells) whose expression was set at 1-fold. GAPDH was used as an endogenous control. Blots are representative of at least three independent experiments. The data are presented as a mean ± SEM of three independent experiments. * represents p ≤ 0.05, compared with the control group. (TIF) [file pone.0163827.s002.tif]

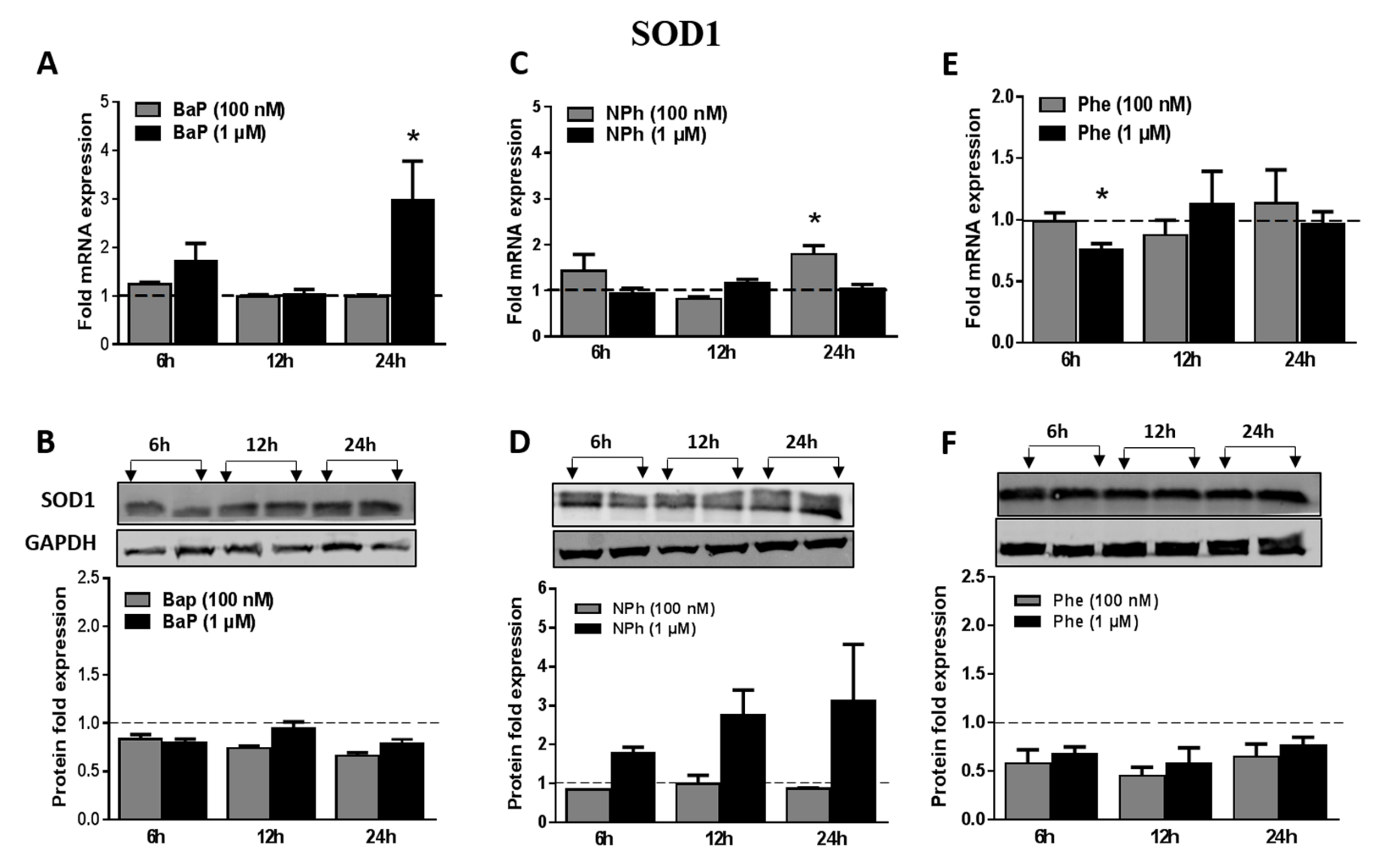

Supplement: S3 Fig — The U937 cells were treated with 100 nM BaP, 100 nM NPh, and 100 nM Phe for 6, 12 and 24 hours. The mRNA fold expressions were calculated using qRT-PCR and the protein fold expressions were quantified by Western blots, and normalized with control (DMSO treated cells) whose expression was set at 1-fold. GAPDH was used as an endogenous control. Blots are representative of at least three independent experiments. The data are presented as a mean ± SEM of three independent experiments. * represents p ≤ 0.05, compared with the control group. (TIF) [file pone.0163827.s003.tif]

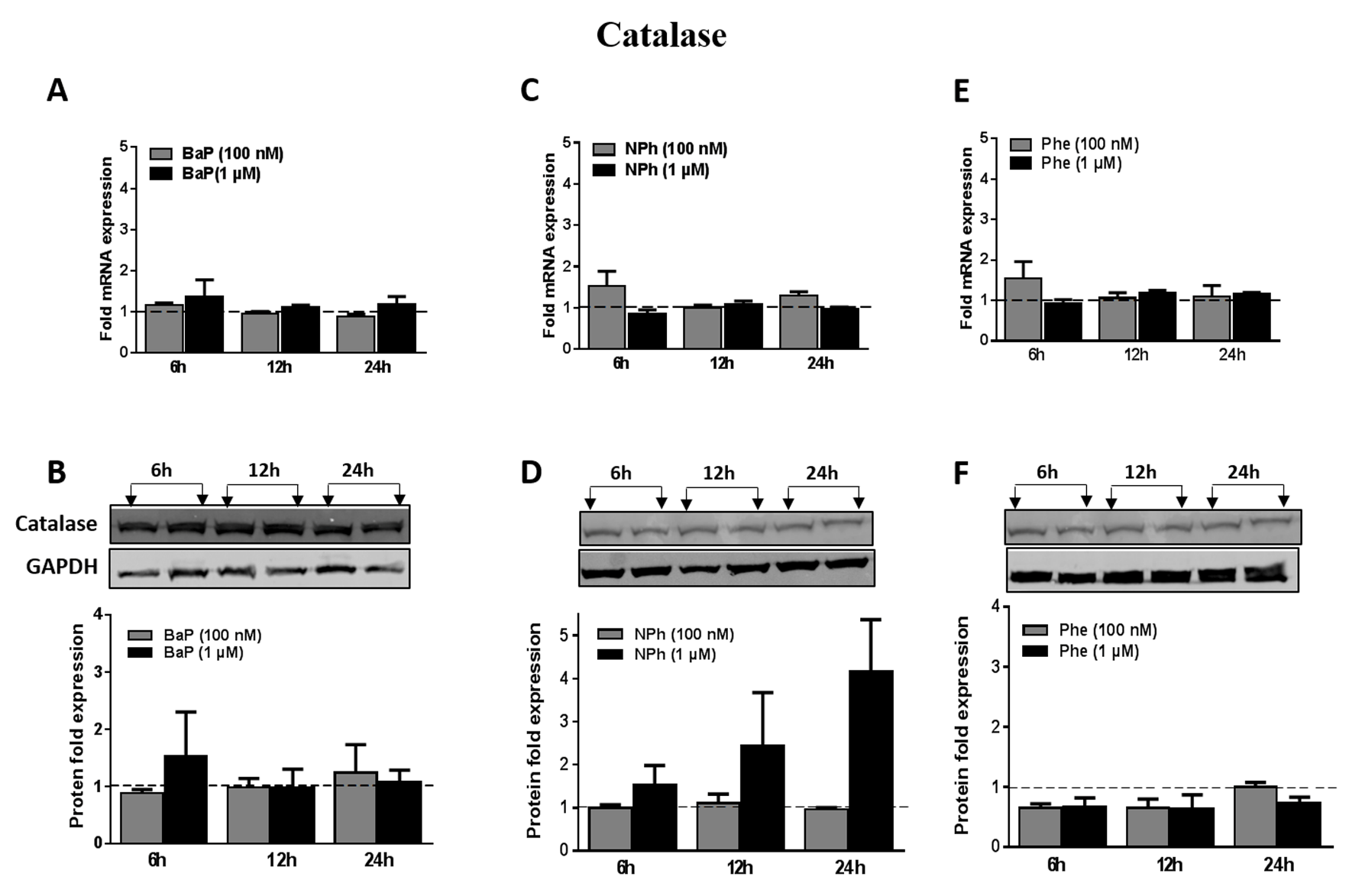

Supplement: S4 Fig — The U937 cells were treated with 100 nM BaP, 100 nM NPh, and 100 nM Phe for 6, 12 and 24 hours. The mRNA fold expressions were calculated using qRT-PCR and the protein fold expressions were quantified by Western blots, and normalized with control (DMSO treated cells) whose expression was set at 1-fold. GAPDH was used as an endogenous control. Blots are representative of at least three independent experiments. The data are presented as a mean ± SEM of three independent experiments. * represents p ≤ 0.05, compared with the control. (TIF) [file pone.0163827.s004.tif]
